# On the Maximum Estrada Index of 3-Uniform Linear Hypertrees

**DOI:** 10.1155/2014/637865

**Published:** 2014-08-28

**Authors:** Faxu Li, Liang Wei, Jinde Cao, Feng Hu, Haixing Zhao

**Affiliations:** ^1^School of Computer Science, Shaanxi Normal University, Xi'an 710062, China; ^2^College of Computer, Qinghai Normal University, Xining 810008, China; ^3^Department of Mathematics, Qinghai Normal University, Xining 810008, China; ^4^Department of Mathematics, Southeast University, Nanjing 210096, China; ^5^Department of Mathematics, Faculty of Science, King Abdulaziz University, Jeddah 21589, Saudi Arabia

## Abstract

For a simple hypergraph *H* on *n* vertices, its Estrada index is defined as $EE(H)=\sum_{i=1}^{n}‍e^{\lambda_{i}}$, where *λ*_1_, *λ*_2_,…, *λ*_*n*_ are the eigenvalues of its adjacency matrix. In this paper, we determine the unique 3-uniform linear hypertree with the maximum Estrada index.

## 1. Introduction

Let *G* = (*V*, *E*) be a simple graph, and let *n* and *m* be the number of vertices and the number of edges of *G*, respectively. The characteristic polynomial of a graph *G* is written as *P*(*G*, *λ*) = det⁡⁡(*λI* − *A*(*G*)), where *A*(*G*) is the adjacency matrix of *G*. The eigenvalues of *G* are the eigenvalues of its adjacency matrix *A*(*G*), which are denoted by *λ*_1_, *λ*_2_,…, *λ*_*n*_. A graph-spectrum-based invariant, nowadays named Estrada index, proposed by Estrada in 2000, is defined as [1]

(1) $$\begin{array}{c} EE\left(G\right)=\overset{n}{\underset{i=1}{\sum }}e^{\lambda_{i}}. \end{array}$$

Since then, the Estrada index has already found remarkable applications in biology, chemistry, and complex networks [2–5]. Some mathematical properties of the Estrada index, especially bounds for it have been established in [6–15]. For more results on the Estrada index, the readers are referred to recent papers [16–19].

Let *H* = (*V*, *E*) be a simple and finite hypergraph with vertex set *V*(*G*) = {*v*_1_, *v*_2_,…, *v*_*n*_} and hyperedge set *E*(*G*) = {*E*_1_, *E*_2_,…, *E*_*m*_}. The hypergraph *H* is called linear if two hyperedges intersect in one vertex at most and also *h*-uniform if |*E*_*i*_ | = *h* for each *E*_*i*_ in *E*, *i* = 1,2,…, *m*. An *h*-uniform hypertree is a connected linear *h*-hypergraph without cycles. An *h*-uniform linear hypertree is called 3-uniform linear hypertree if *h* is equal to 3. Denoted by *S*_*m*_^*h*^ an *h*-uniform linear star with *m* hyperedges. More details on hypergraphs can be found in [20].

Let *A*(*H*) denote a square symmetric matrix in which the diagonal elements *a*_*ij*_ are zero, and other elements *a*_*ij*_ represent the number of hyperedges containing both vertices *v*_*i*_ and *v*_*j*_ (for undirected hypergraphs, *a*_*ij*_ = *a*_*ji*_). Let *λ*_1_, *λ*_2_,…, *λ*_*n*_ be the eigenvalues of *A*(*H*) of *H*. The subhypergraph centrality of a hypergraph *H*, firstly put forward by Estrada and Rodríguez-Velázquez in 2006, is defined as [21]

(2) $$\begin{array}{c} \langle C_{SH}\rangle =\frac{1}{n}\overset{n}{\underset{i=1}{\sum }}C_{SH}\left(i\right)=\frac{1}{n}\overset{n}{\underset{i=1}{\sum }}e^{\lambda_{i}}. \end{array}$$

They revealed that the subhypergraph centrality provides a measure of the centrality of complex hypernetworks (social, reaction, metabolic, protein, food web, etc). For convenience, we call the subhypergraph centrality of a hypergraph its Estrada index and define the Estrada index as

(3) $$\begin{array}{c} EE\left(H\right)=\overset{n}{\underset{i=1}{\sum }}e^{\lambda_{i}}. \end{array}$$

Thus far, results on the Estrada index of hypergraph seem to be few although the Estrada index of graph has numerous applications. So our main goal is to investigate the Estrada index of 3-uniform linear hypertrees. In this paper, we determine the unique 3-uniform linear hypertree with the maximum Estrada index among the set of 3-uniform linear hypertrees.

## 2. Preliminaries

**Figure 1:**
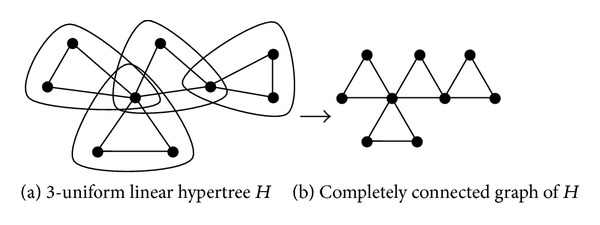
A 3-uniform linear hypertree *H* and its completely connected graph *G*_*H*_.

For a hypergraph *H* of order *n*, its completely connected graph, denoted by *G*_*H*_, is a graph which has the same order and in which two vertices are adjacent if they share one hyperedge. Obviously, *G*_*H*_ is a multigraph. For an *h*-uniform linear hypergraph *H*, *G*_*H*_ is a simple graph. According to the definition of adjacency matrix of hypergraph, it is easy to see that both a 3-uniform linear hypertree *H* and its completely connected graph *G*_*H*_ have the same adjacency matrix; see Figure 1. Then, they have the identical Estrada index. Thus, we investigate the Estrada index of its completely connected graphs instead of the 3-uniform linear hypertrees in this paper.

We use *M*_*k*_(*G*) = ∑_*i*=1_^*n*^ *λ*_*i*_^*k*^ to denote the *k*th spectral moment of the graph *G*. It is well-known [22] that *M*_*k*_(*G*) is equal to the number of closed walks of length *k* in *G*. Obviously, for any graph *G*, *M*_0_(*G*) = *n*, *M*_1_(*G*) = 0, *M*_2_(*G*) = 2*m*, *M*_3_(*G*) = 6*t*, and *M*_4_(*G*) = 2∑_*i*=1_^*n*^ *d*_*i*_^2^ − 2*m* + 8*q*, where *t*, *q*, and *d*_*i*_ = *d*_*G*_(*v*_*i*_) are the number of triangles, the number of quadrangles, and the degree of vertex *v*_*i*_ in graph *G*, respectively. Then

(4) $$\begin{array}{c} EE\left(G\right)=\overset{n}{\underset{i=1}{\sum }}\overset{\infty }{\underset{k=0}{\sum }}\frac{\lambda_{i}^{k}}{k!}=\overset{\infty }{\underset{k=0}{\sum }}\frac{M_{k}\left(G\right)}{k!}. \end{array}$$

For *u*, *v* ∈ *V*(*G*), denote by *W*_*k*_(*G*; *u*, *v*) the set of (*u*, *v*)-walks of length *k* in *G*. Obviously, *M*_*k*_(*G*; *u*, *v*) = |*W*_*k*_(*G*; *u*, *v*)|. For convenience, let *W*_*k*_(*G*; *u*) = *W*_*k*_(*G*; *u*, *u*) and *M*_*k*_(*G*; *u*) = *M*_*k*_(*G*; *u*, *u*). Let *W* be a (*u*, *v*)-walk in graph *G*; we denote by *W* ^−1^ a (*v*, *u*)-walk obtained from *W* by reversing *W*.

For any two graphs *G*_1_ and *G*_2_, if *M*_*k*_(*G*_1_) ≥ *M*_*k*_(*G*_2_) for all integers *k* > 0, then *EE*(*G*_1_) ≥ *EE*(*G*_2_). Moreover, if the strict inequality *M*_*k*_(*G*_1_) > *M*_*k*_(*G*_2_) holds for at least one value *k* > 0, then *EE*(*G*_1_) > *EE*(*G*_2_).

Denote by Γ(*n*, *m*) the set of connected graphs on *n* vertices and *m* triangles such that any two triangles have a common vertex at most. Apparently, for a 3-uniform linear hypertree *H* on *n* vertices and *m* hyperedges, *G*_*H*_ ∈ Γ(*n*, *m*). Now we study the Estrada index of a graph in Γ(*n*, *m*).

## 3. Maximum Estrada Index of 3-Uniform Linear Hypertrees

In this section, we determine the maximum value of Estrada index among the set of 3-uniform linear hypertrees.

**Lemma 1 .**

**Figure 2:**
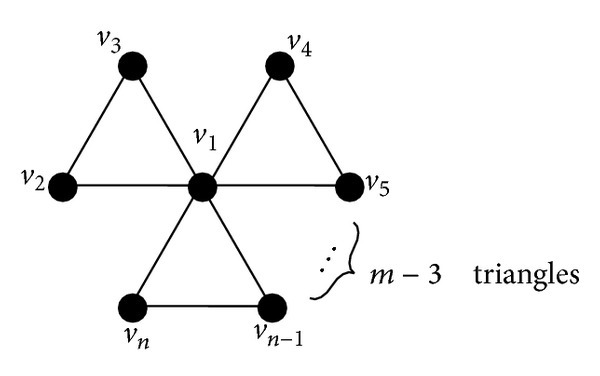
The star *S*_*m*_^3^ on *m* triangles.

Let *S*_*m*_^3^ be star which is the completely connected graph of *S*_*m*_^3^ with *m* hyperedges. It is easily found that the star *S*_*m*_^3^ has *n* vertices labled *v*_1_, *v*_2_, *v*_3_,…, *v*_*n*_ and *m* = (*n* − 1)/2 triangles. Let *k* be a positive integer; then there is an injection *ξ* from *W*_*k*_(*S*_*m*_^3^; *v*_2_) to *W*_*k*_(*S*_*m*_^3^; *v*_1_), and *ξ* is not surjective for *n* ≥ 5, 2 ≤ *m* ≤ (*n* − 1)/2, and *k* > 1, where *W*_*k*_(*S*_*m*_^3^; *v*_2_) and *W*_*k*_(*S*_*m*_^3^; *v*_1_) are the sets of closed walks of length *k* of *v*_2_ and *v*_1_ in *S*_*m*_^3^, respectively; see Figure 2.

**Proof**

Firstly, we construct a mapping *φ* from *W*_*k*_(*S*_*m*_^3^; *v*_2_) to *W*_*k*_(*S*_*m*_^3^; *v*_1_). For *W* ∈ *W*_*k*_(*S*_*m*_^3^; *v*_2_), let *φ*(*W*) be the closed walk obtained from *W* by replacing *v*_1_ by *v*_2_ and *v*_2_ by *v*_1_. Obviously, *φ*(*W*) ∈ *W*_*k*_(*S*_*m*_^3^; *v*_1_) and *φ* is a bijection.

Secondly, we construct a mapping *ξ* from *W*_*k*_(*S*_*m*_^3^; *v*_2_) to *W*_*k*_(*S*_*m*_^3^; *v*_1_). For *W* ∈ *W*_*k*_(*S*_*m*_^3^; *v*_2_), we consider the following cases.

*Case  1.* Suppose *W* does not pass the edge *v*_1_ *v*_*t*_ for *t* ≥ 4; then *ξ*(*W*) = *φ*(*W*).

*Case  2.* Suppose *W* passes the edge *v*_1_ *v*_*t*_ for *t* ≥ 4. For *W* ∈ *W*_*k*_(*S*_*m*_^3^; *v*_2_), we may uniquely decompose *W* into three sections *W*_1_ *W*_2_ *W*_3_, where *W*_1_ is the longest (*v*_2_, *v*_1_)-section of *W* without *v*_*t*_, *W*_2_ is the internal longest (*v*_*t*_, *v*_*t*_′)-section of *W* for *t*′ ≥ 4, and the last *W*_3_ is the remaining (*v*_1_, *v*_2_)-section of *W* not containing *v*_*t*_. We consider the following three subcases.

*Case  2.1.* If both *W*_1_ and *W*_3_ contain the vertex *v*_3_, we may uniquely decompose *W*_1_ into two sections *W*_11_ *W*_12_ and decompose *W*_3_ into two sections *W*_31_ *W*_32_, where *W*_11_ is the shortest (*v*_2_, *v*_3_)-section of *W*_1_, *W*_12_ is the remaining (*v*_3_, *v*_1_)-section of *W*_1_, *W*_31_ is the longest (*v*_1_, *v*_3_)-section of *W*_3_, and *W*_32_ is the remaining (*v*_3_, *v*_2_)-section of *W*_3_.

Let *ξ*(*W*) = *ξ*(*W*_11_)*ξ*(*W*_12_)*ξ*(*W*_2_)*ξ*(*W*_31_)*ξ*(*W*_32_), where *ξ*(*W*_12_) = *W*_12_, *ξ*(*W*_2_) = *W*_2_, *ξ*(*W*_31_) = *W*_31_, *ξ*(*W*_11_) is a (*v*_1_, *v*_3_)-walk obtained from *W*_11_ replacing *v*_1_ by *v*_2_ and *v*_2_ by *v*_1_, and *ξ*(*W*_32_) is a (*v*_3_, *v*_1_)-walk obtained from *W*_32_ replacing *v*_1_ by *v*_2_ and *v*_2_ by *v*_1_.

*Case  2.2.* If *W*_1_ contains the vertex *v*_3_ and *W*_3_ does not contain *v*_3_, let *ξ*(*W*) = *ξ*(*W*_1_)*ξ*(*W*_2_)*ξ*(*W*_3_), where *ξ*(*W*_2_) = *W*_2_, *ξ*(*W*_1_) is a (*v*_1_, *v*_1_)-walk obtained from *W*_1_ replacing its first vertex *v*_2_ by *v*_1_ *v*_2_, and *ξ*(*W*_3_) is a (*v*_1_, *v*_1_)-walk obtained from *W*_3_ replacing its last two vertices *v*_1_ *v*_2_ by *v*_1_.

*Case  2.3.* If *W*_1_ does not contain the vertex *v*_3_, let *ξ*(*W*) = *ξ*(*W*_1_)*ξ*(*W*_2_)*ξ*(*W*_3_), where *ξ*(*W*_2_) = *W*_2_, *ξ*(*W*_1_) is a (*v*_1_, *v*_1_)-walk obtained from *W*_1_ replacing its first two vertices *v*_2_ *v*_1_ by *v*_1_, and *ξ*(*W*_3_) is a (*v*_1_, *v*_1_)-walk obtained from *W*_3_ replacing its last vertex *v*_2_ by *v*_2_ *v*_1_.

For example, in star *S*_3_^3^ on 7 vertices and 3 triangles, *W* = *v*_2_ *v*_3_ *v*_1_ *v*_2_ *v*_1_ *v*_3_ *v*_2_ is a closed walk of length 6 of *v*_2_ not passing the edge *v*_1_ *v*_*t*_. By Case  1, we have

(5) $$\begin{array}{c} \xi \left(W\right)=v_{1}v_{3}v_{2}v_{1}v_{2}v_{3}v_{1}. \end{array}$$

*W*′ = *v*_2_ *v*_3_ *v*_1_ *v*_4_ *v*_5_ *v*_1_ *v*_6_ *v*_7_ *v*_1_ *v*_2_ is a closed walk of length 9 of *v*_2_ passing the edge *v*_1_ *v*_*t*_. By Case*  *2.2, we get

(6) $$\begin{array}{c} \xi \left(W^{\prime }\right)=v_{1}v_{2}v_{3}v_{1}v_{4}v_{5}v_{1}v_{6}v_{7}v_{1}. \end{array}$$

*W*′′ = *v*_2_ *v*_1_ *v*_2_ *v*_1_ *v*_4_ *v*_5_ *v*_1_ *v*_2_ *v*_3_ *v*_1_ *v*_6_ *v*_7_ *v*_1_ *v*_3_ *v*_2_ is a closed walk of length 14 of *v*_2_ passing the edge *v*_1_ *v*_*t*_. By Case*  *2.3, we obtain

(7) $$\begin{array}{c} \xi \left(W^{\prime \prime }\right)=v_{1}v_{2}v_{1}v_{4}v_{5}v_{1}v_{2}v_{3}v_{1}v_{6}v_{7}v_{1}v_{3}v_{2}v_{1}. \end{array}$$

Obviously, *ξ*(*W*) ∈ *W*_*k*_(*S*_*m*_^3^; *v*_1_), *ξ* is an injective and not a surjective for *n* ≥ 5, and *k* ≥ 1.

**Lemma 2 .**

**Figure 3:**
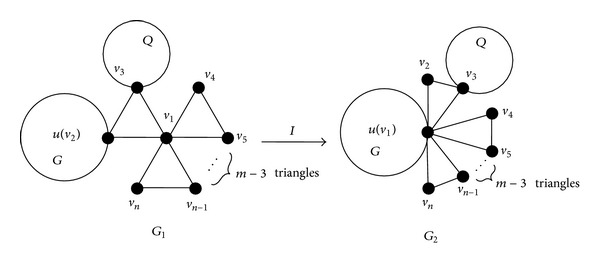
Transformation *I*.

Let *u* be a nonisolated vertex of a connected graph *G*. If *G*_1_ and *G*_2_ are the graphs obtained from *G* by identifying an external vertex *v*_2_ and the center vertex *v*_1_ of the union of *S*_*m*_^3^ ∪ *Q* to *u*, respectively, where |*V*(*S*_*m*_^3^)| = *n*, *Q* is either empty graph or nonempty graph. Then *M*_*k*_(*G*_1_) < *M*_*k*_(*G*_2_) for *n* ≥ 5 and *k* ≥ 4; see Figure 3.

**Proof**

Let *W*_*k*_(*G*_*i*_)  (*W*_*k*_(*G*), *W*_*k*_(*S*_*m*_^3^ ∪ *Q*), resp.) be the set of closed walks of length *k* of *G*_*i*_(*G*, *S*_*m*_^3^ ∪ *Q*, resp.) for *i* = 1,2. Then *W*_*k*_(*G*_*i*_) = *W*_*k*_(*G*) ∪ *W*_*k*_(*S*_*m*_^3^ ∪ *Q*) ∪ *X*_*i*_ is a partition, where *X*_*i*_ is the set of closed walks of length *k* of *G*_*i*_; each of them contains both at least one edge in *E*(*G*) and at least one edge in *E*(*S*_*m*_^3^ ∪ *Q*). So *M*_*k*_(*G*_*i*_) = |*W*_*k*_(*G*)|+|*W*_*k*_(*S*_*m*_^3^ ∪ *Q*)|+|*X*_*i*_ | = *M*_*k*_(*G*) + *M*_*k*_(*S*_*m*_^3^ ∪ *Q*)+|*X*_*i*_|. Thus we need to show the inequality |*X*_1_ | <|*X*_2_|.

We construct a mapping *η* from *X*_1_ to *X*_2_ and consider the following four cases.

*Case  1.* Suppose *W* is a closed walk starting from *u* ∈ *V*(*G*) in *X*_1_. For *W* ∈ *X*_1_, let *η*(*W*) = (*W* − *W*∩(*S*_*m*_^3^ ∪ *Q*)) ∪ *ξ*(*W*∩(*S*_*m*_^3^ ∪ *Q*)); that is, *η*(*W*) is the closed walk in *X*_2_ obtained from *W* by replacing its every section in *S*_*m*_^3^ ∪ *Q* with its image under the map *ξ*.

*Case  2.* Suppose *W* is a closed walk starting at *v*_1_ in *X*_1_. For *W* ∈ *X*_1_, we may uniquely decompose *W* into three sections *W*_1_ *W*_2_ *W*_3_, where *W*_1_ is the longest (*v*_1_, *v*_2_)-section of *W* without vertices *u*_0_,…, *u*_*t*_′′ ∈ *V*(*G*), *W*_2_ is the internal longest (*u*_0_, *u*_*t*_′′)-section of *W* (for which the internal vertices are some possible vertices in *V*(*G*_1_)), and *W*_3_ is the remaining (*v*_2_, *v*_1_)-section of *W*. Let *η*(*W*) = *η*(*W*_1_)*η*(*W*_2_)*η*(*W*_3_), where *η*(*W*_1_) = *W*_1_^−1^, *η*(*W*_3_) = *W*_3_^−1^, and *η*(*W*_2_) = (*W*_2_ − *W*_2_∩(*S*_*m*_^3^ ∪ *Q*)) ∪ *ξ*(*W*_2_∩(*S*_*m*_^3^ ∪ *Q*)); that is, *η*(*W*_2_) is a (*u*_0_, *u*_*t*_′′)-walk from *W*_2_ by replacing its every section in *S*_*m*_^3^ ∪ *Q* with its image under the map *ξ*.

*Case  3.* Suppose *W* is a closed walk starting from *v*_3_ or *w* ∈ *V*(*Q*) in *X*_1_. For *W* ∈ *X*_1_, we may uniquely decompose *W* into three sections *W*_1_ *W*_2_ *W*_3_, where *W*_1_ is the longest (*v*_3_, *v*_2_) (or (*w*, *v*_2_))-section of *W* without vertices *u*_0_,…, *u*_*t*_′′, *W*_2_ is the internal longest (*u*_0_, *u*_*t*_′′)-section of *W* (for which the internal vertices are some possible vertices in *V*(*G*_1_)), and *W*_3_ is the remaining (*v*_2_, *v*_3_) (or (*v*_2_, *w*))-section of *W* without vertices *u*_0_,…, *u*_*t*_′′. We have three subcases.

*Case  3.1*. If both *W*_1_ and *W*_3_ do not pass edge *v*_1_ *v*_*t*_, let *η*(*W*) = *η*(*W*_1_)*η*(*W*_2_)*η*(*W*_3_), where *η*(*W*_2_) = (*W*_2_ − *W*_2_∩(*S*_*m*_^3^ ∪ *Q*)) ∪ *ξ*(*W*_2_∩(*S*_*m*_^3^ ∪ *Q*)), *η*(*W*_1_) is a (*v*_3_, *v*_1_) (or (*w*, *v*_1_))-walk obtained from *W*_1_ replacing *v*_1_ by *v*_2_ and *v*_2_ by *v*_1_, and *η*(*W*_3_) is a (*v*_1_, *v*_3_) (or (*v*_1_, *w*))-walk obtained from *W*_3_ replacing *v*_1_ by *v*_2_ and *v*_2_ by *v*_1_.

*Case  3.2*. If both *W*_1_ and *W*_3_ pass edge *v*_1_ *v*_*t*_, we may anew decompose *W* into five sections *W*_1_ *W*_2_ *W*_3_ *W*_4_ *W*_5_, where *W*_1_ is the longest (*v*_3_, *v*_*t*_) (or (*w*, *v*_*t*_))-section of *W* (which do not contain vertices *u*_0_,…, *u*_*t*_′′), *W*_2_ is the second (*v*_1_, *v*_2_)-section of *W* (for which the internal vertices, if exist, are only possible *v*_1_, *v*_2_, *v*_3_, *w* ∈ *V*(*Q*)), the third *W*_3_ is the internal longest (*u*_0_, *u*_*t*_′′)-section of *W* (for which the internal vertices are some possible vertices in *V*(*G*_1_)), the fourth *W*_4_ is the longest (*v*_2_, *v*_1_)-section of *W* (for which the internal vertices, if exist, are only possible *v*_1_, *v*_2_, *v*_3_, *w* ∈ *V*(*Q*)), and the last *W*_5_ is the remaining (*v*_*t*_′, *v*_3_) (or (*v*_*t*_, *w*))-section of *W*. We have three subsubcases.

*Case  3.2.1.* If both *W*_2_ and *W*_4_ contain the vertex *v*_3_, we may uniquely decompose *W*_2_ into two sections *W*_21_ *W*_22_ and *W*_4_ into two sections *W*_41_ *W*_42_, where *W*_21_ is the longest (*v*_1_, *v*_3_)-section of *W*_2_, *W*_22_ is the remaining shortest (*v*_3_, *v*_2_) of *W*_2_, *W*_41_ is the shortest (*v*_2_, *v*_3_)-section of *W*_4_, and *W*_42_ is the remaining longest (*v*_3_, *v*_1_)-section of *W*_4_.

Let *η*(*W*) = *η*(*W*_1_)*η*(*W*_21_)*η*(*W*_22_)*η*(*W*_3_)*η*(*W*_41_)*η*(*W*_42_)*η*(*W*_5_), where *η*(*W*_1_) = *W*_1_, *η*(*W*_21_) = *W*_21_, *η*(*W*_3_) = (*W*_3_ − *W*_3_∩(*S*_*m*_^3^ ∪ *Q*)) ∪ *ξ*(*W*_3_∩(*S*_*m*_^3^ ∪ *Q*)), *η*(*W*_42_) = *W*_42_ *η*(*W*_5_) = *W*_5_, *η*(*W*_22_) is a (*v*_3_, *v*_1_)-walk obtained from *W*_22_ replacing *v*_1_ by *v*_2_ and *v*_2_ by *v*_1_, and *η*(*W*_41_) is a (*v*_1_, *v*_3_)-walk obtained from *W*_41_ replacing *v*_1_ by *v*_2_ and *v*_2_ by *v*_1_.

*Case  3.2.2*. If *W*_2_ does not contain the vertex *v*_3_, let *η*(*W*) = *η*(*W*_1_)*η*(*W*_2_)*η*(*W*_3_)*η*(*W*_4_)*η*(*W*_5_), where *η*(*W*_1_) = *W*_1_, *η*(*W*_3_) = (*W*_3_ − *W*_3_∩(*S*_*m*_^3^ ∪ *Q*)) ∪ *ξ*(*W*_3_∩(*S*_*m*_^3^ ∪ *Q*)), *η*(*W*_5_) = *W*_5_, *η*(*W*_2_) is a (*v*_1_, *v*_1_)-walk obtained from *W*_2_ replacing its last two vertices *v*_1_ *v*_2_ by *v*_1_, and *η*(*W*_4_) is a (*v*_1_, *v*_1_)-walk obtained from *W*_4_ replacing its first vertex *v*_2_ by *v*_1_ *v*_2_.

*Case  3.2.3.* If *W*_2_ contains the vertex *v*_3_ and *W*_4_ does not contain vertex *v*_3_, let *η*(*W*) = *η*(*W*_1_)*η*(*W*_2_)*η*(*W*_3_)*η*(*W*_4_)*η*(*W*_5_), where *η*(*W*_1_) = *W*_1_, *η*(*W*_3_) = (*W*_3_ − *W*_3_∩(*S*_*m*_^3^ ∪ *Q*)) ∪ *ξ*(*W*_3_∩(*S*_*m*_^3^ ∪ *Q*)), *η*(*W*_5_) = *W*_5_, *η*(*W*_2_) is a (*v*_1_, *v*_1_)-walk obtained from *W*_2_ replacing its last vertex *v*_2_ by *v*_2_ *v*_1_, and *η*(*W*_4_) is a (*v*_1_, *v*_1_)-walk obtained from *W*_4_ replacing its first two vertices *v*_2_ *v*_1_ by *v*_1_.

*Case  3.3.* If *W*_1_ passes edge *v*_1_ *v*_*t*_ and *W*_3_ does not pass edge *v*_1_ *v*_*t*_, we may anew decompose *W* into four sections *W*_1_ *W*_2_ *W*_3_ *W*_4_, where *W*_1_ is the longest (*v*_3_, *v*_*t*_) (or (*w*, *v*_*t*_))-section of *W* (which do not contain vertices *u*_0_,…, *u*_*t*_′′), *W*_2_ is the second (*v*_1_, *v*_2_)-section of *W* (for which the internal vertices, if exist, are only possible *v*_1_, *v*_2_, *v*_3_, *w* ∈ *V*(*Q*)), the third *W*_3_ is the internal longest (*u*_0_, *u*_*t*_′′)-section of *W* (for which the internal vertices are some possible vertices in *V*(*G*_1_)), and the last *W*_4_ is the longest (*v*_2_, *v*_3_) (or (*v*_2_, *w*))-section of *W* (for which the internal vertices, if exist, are only possible *v*_1_, *v*_2_, *v*_3_, *w* ∈ *V*(*Q*)). We consider the following two subsubcases.

*Case  3.3.1.* If *W*_2_ contains vertex *v*_3_, we may uniquely decompose *W*_2_ into two sections *W*_21_ *W*_22_, where *W*_21_ is the longest (*v*_1_, *v*_3_)-section of *W*_2_ and *W*_22_ is the remaining shortest (*v*_3_, *v*_2_)-section of *W*_2_.

Let *η*(*W*) = *η*(*W*_1_)*η*(*W*_21_)*η*(*W*_22_)*η*(*W*_3_)*η*(*W*_4_), where *η*(*W*_1_) = *W*_1_, *η*(*W*_21_) = *W*_21_, *η*(*W*_3_) = (*W*_3_ − *W*_3_∩(*S*_*m*_^3^ ∪ *Q*)) ∪ *ξ*(*W*_3_∩(*S*_*m*_^3^ ∪ *Q*)), *η*(*W*_22_) is a (*v*_3_, *v*_1_)-walk obtained from *W*_22_ replacing *v*_1_ by *v*_2_ and *v*_2_ by *v*_1_, and *η*(*W*_4_) is a (*v*_1_, *v*_3_) (or (*v*_1_, *w*))-walk obtained from *W*_4_ replacing *v*_1_ by *v*_2_ and *v*_2_ by *v*_1_.

*Case  3.3.2*. If *W*_2_ does not contain vertex *v*_3_, let *η*(*W*) = *η*(*W*_1_)*η*(*W*_2_)*η*(*W*_3_)*η*(*W*_4_), where *η*(*W*_1_) = *W*_1_, *η*(*W*_3_) = (*W*_3_ − *W*_3_∩(*S*_*m*_^3^ ∪ *Q*)) ∪ *ξ*(*W*_3_∩(*S*_*m*_^3^ ∪ *Q*)), *η*(*W*_2_) is a (*v*_1_, *v*_1_)-walk obtained from *W*_2_ replacing its last two vertices *v*_1_ *v*_2_ by *v*_1_, and *η*(*W*_4_) is a (*v*_1_, *v*_3_) (or (*v*_1_, *w*))-walk obtained from *W*_4_ replacing its first vertex *v*_2_ by *v*_1_ *v*_2_.

*Case  3.4*. If *W*_1_ does not pass edge *v*_1_ *v*_*t*_ and *W*_3_ passes edge *v*_1_ *v*_*t*_, we may anew decompose *W* into four sections *W*_1_ *W*_2_ *W*_3_ *W*_4_, where *W*_1_ is the longest (*v*_3_, *v*_2_) (or (*w*, *v*_2_))-section of *W* (which do not contain vertices *u*_0_,…, *u*_*t*_′′ and must contain vertex *v*_3_), the second *W*_2_ is the internal longest (*u*_0_, *u*_*t*_′′)-section of *W* (for which the internal vertices are some possible vertices in *V*(*G*_1_)), *W*_3_ is the third (*v*_2_, *v*_1_)-section of *W* (for which the internal vertices, if exist, are only possible *v*_1_, *v*_2_, *v*_3_, *w* ∈ *V*(*Q*)), and the last *W*_4_ is the longest (*v*_*t*_, *v*_3_) (or (*v*_*t*_, *w*))-section of *W*. We have two subsubcases.

*Case  3.4.1.* If *W*_3_ contains vertex *v*_3_, we may uniquely decompose it into two sections *W*_31_ *W*_32_, where *W*_31_ is the shortest (*v*_2_, *v*_3_)-section of *W*_3_ and *W*_32_ is the remaining longest (*v*_3_, *v*_1_)-section of *W*_3_.

Let *η*(*W*) = *η*(*W*_1_)*η*(*W*_2_)*η*(*W*_31_)*η*(*W*_32_)*η*(*W*_4_), where *η*(*W*_2_) = (*W*_2_ − *W*_2_∩(*S*_*m*_^3^ ∪ *Q*)) ∪ *ξ*(*W*_2_∩(*S*_*m*_^3^ ∪ *Q*)), *η*(*W*_32_) = *W*_32_, *η*(*W*_4_) = *W*_4_, *η*(*W*_1_) is a (*v*_3_, *v*_1_) (or (*w*, *v*_1_))-walk obtained from *W*_1_ replacing *v*_1_ by *v*_2_ and *v*_2_ by *v*_1_, and *η*(*W*_31_) is a (*v*_1_, *v*_3_)-walk obtained from *W*_31_ replacing *v*_1_ by *v*_2_ and *v*_2_ by *v*_1_.

*Case  3.4.2.* If *W*_3_ does not contain vertex *v*_3_, let *η*(*W*) = *η*(*W*_1_)*η*(*W*_2_)*η*(*W*_3_)*η*(*W*_4_), where *η*(*W*_2_) = (*W*_2_ − *W*_2_∩*S*_*m*_^3^) ∪ *ξ*(*W*_2_∩*S*_*m*_^3^), *η*(*W*_4_) = *W*_4_, *η*(*W*_1_) is a (*v*_3_, *v*_1_) (or (*w*, *v*_1_))-walk obtained from *W*_1_ replacing its last vertex *v*_2_ by *v*_2_ *v*_1_, and *η*(*W*_3_) is a (*v*_1_, *v*_1_)-walk obtained from *W*_3_ replacing its first two vertices *v*_2_ *v*_1_ by *v*_1_.

*Case  4*. Suppose *W* is a closed walk starting from *v*_*i*_ for *i* = 4,5, 6,…, *n* in *X*_1_. For *W* ∈ *X*_1_, we may uniquely decompose *W* into five sections *W*_1_ *W*_2_ *W*_3_ *W*_4_ *W*_5_, where *W*_1_ is the longest (*v*_*i*_, *v*_*t*_)-section of *W* (which do not contain vertices *u*_0_,…, *u*_*t*_′′), *W*_2_ is the second (*v*_1_, *v*_2_)-section of *W* (for which the internal vertices, if exist, are only possible *v*_1_, *v*_2_, *v*_3_, *w* ∈ *V*(*G*)), the third *W*_3_ is the internal longest (*u*_0_, *u*_*t*_′′)-section of *W* (for which the internal vertices are some possible vertices in *V*(*G*_1_)), the fourth *W*_4_ is the longest (*v*_2_, *v*_1_)-section of *W* (for which the internal vertices, if exist, are only possible *v*_1_, *v*_2_, *v*_3_, *w* ∈ *V*(*G*)), and the last *W*_5_ is the remaining (*v*_*t*_′, *v*_*i*_)-section of *W*. We have four subcases.

*Case  4.1*. If both *W*_2_ and *W*_4_ contain the vertex *v*_3_, we may uniquely decompose *W*_2_ into two sections *W*_21_ *W*_22_ and decompose *W*_4_ into two sections *W*_41_ *W*_42_, where *W*_21_ is the longest (*v*_1_, *v*_3_)-section of *W*_2_, *W*_22_ is the remaining shortest (*v*_3_, *v*_2_) of *W*_2_, *W*_41_ is the shortest (*v*_2_, *v*_3_)-section of *W*_4_, and *W*_42_ is the remaining longest (*v*_3_, *v*_1_)-section of *W*_4_.

Let *η*(*W*) = *η*(*W*_1_)*η*(*W*_21_)*η*(*W*_22_)*η*(*W*_3_)*η*(*W*_41_)*η*(*W*_42_)*η*(*W*_5_), where *η*(*W*_1_) = *W*_1_, *η*(*W*_21_) = *W*_21_, *η*(*W*_3_) = (*W*_3_ − *W*_3_∩(*S*_*m*_^3^ ∪ *Q*)) ∪ *ξ*(*W*_3_∩(*S*_*m*_^3^ ∪ *Q*)), *η*(*W*_42_) = *W*_42_ *η*(*W*_5_) = *W*_5_, *η*(*W*_22_) is a (*v*_3_, *v*_1_)-walk obtained from *W*_22_ replacing *v*_1_ by *v*_2_ and *v*_2_ by *v*_1_, and *η*(*W*_41_) is a (*v*_1_, *v*_3_)-walk obtained from *W*_41_ replacing *v*_1_ by *v*_2_ and *v*_2_ by *v*_1_.

*Case  4.2*. If *W*_2_ contains the vertex *v*_3_ and *W*_4_ does not contain vertex *v*_3_, let *η*(*W*) = *η*(*W*_1_)*η*(*W*_2_)*η*(*W*_3_)*η*(*W*_4_)*η*(*W*_5_), where *η*(*W*_1_) = *W*_1_, *η*(*W*_3_) = (*W*_3_ − *W*_3_∩(*S*_*m*_^3^ ∪ *Q*)) ∪ *ξ*(*W*_3_∩(*S*_*m*_^3^ ∪ *Q*)), *η*(*W*_5_) = *W*_5_, *η*(*W*_2_) is a (*v*_1_, *v*_1_)-walk obtained from *W*_2_ replacing its last vertex *v*_2_ by *v*_2_ *v*_1_, and *η*(*W*_4_) is a (*v*_1_, *v*_1_)-walk obtained from *W*_4_ replacing its first two vertices *v*_2_ *v*_1_ by *v*_1_.

*Case  4.3*. If *W*_2_ does not contain the vertex *v*_3_, let *η*(*W*) = *η*(*W*_1_)*η*(*W*_2_)*η*(*W*_3_)*η*(*W*_4_)*η*(*W*_5_), where *η*(*W*_1_) = *W*_1_, *η*(*W*_3_) = (*W*_3_ − *W*_3_∩(*S*_*m*_^3^ ∪ *Q*)) ∪ *ξ*(*W*_3_∩(*S*_*m*_^3^ ∪ *Q*)), *η*(*W*_5_) = *W*_5_, *η*(*W*_2_) is a (*v*_1_, *v*_1_)-walk obtained from *W*_2_ by replacing its last two vertices *v*_1_ *v*_2_ by *v*_1_, and *η*(*W*_4_) is a (*v*_1_, *v*_1_)-walk obtained from *W*_4_ by replacing its first vertex *v*_2_ by *v*_1_ *v*_2_.

For example,

(8) η1(u0u1⋯urv2v3w1⋯wtv3v1v2u1′⋯us′v2v1v3v2u1′′⋯ut′′u0)=u0u1⋯urv1v3w1⋯wtv3v2v1u1′⋯ us′v1v2v3v1u1′′⋯ut′′u0,η1(u0u1⋯urv2v3w1⋯wtv3v1v4v5v1v2u1′⋯us′v2v3v5v4v1v3v2u1′′⋯ut′′u0)=u0u1⋯urv1v2v3w1⋯wtv3v1v4v5v1u1′ ⋯us′v1v3v5v4v1v3v1u1′′⋯ut′′u0,η1(v3w1⋯wtv3v2u1⋯urv2v1v5v4v1v2u1′⋯us′v2v1v3)=v3w1⋯wtv3v1u1⋯urv1v5v4v1v2v1u1′⋯us′v1v2v3,η1(v3v2v1v7v6v1v2v1v2u1⋯urv2v1v4v5v1v3v2u1′⋯  us′v2v1v7v6v1v2v3)=v3v2v1v7v6v1v2v1u1⋯urv1v4v5v1v3v2v1u1′ ⋯us′v1v2v1v7v6v1v2v3,η1(v1v4v5v1v3v2u1⋯urv2v3v2v1v4v5v1v3v2u1′⋯us′v2v1)=v2v3v1v5v4v1u1⋯urv1v3v2v1v4v5v1v3v1u1′⋯us′v1v2,η1(v4v5v1v3v2u1⋯urv2v3v2v1v4v5v1v3v2u1′  ⋯us′v2v3v2v1v5v4)=v4v5v1v3v1u1⋯urv1v3v2v1v4v5v1v3v1u1′ ⋯us′v1v3v2v1v5v4,

where *u*_0_, *u*_1_,…, *u*_*r*_, *u*_1_′_ _,…, *u*_*s*_′_ _, *u*_1_′′,…, *u*_*t*_′′ are vertices in *G* and *w*_1_,…, *w*_*t*_ are vertices in *Q*.

By Lemma 1, *ξ* is injective and not surjective. It is easily shown that *η* is also injective and not surjective. Thus |*X*_1_ | <|*X*_2_|, *M*_*k*_(*G*_1_) < *M*_*k*_(*G*_2_).

**Theorem 3 .**

Let *G*_*H*_ be an arbitrary graph on *n* vertices in set Γ(*n*, *m*), where *n* > 5. Then *EE*(*G*_*H*_) ≤ *EE*(*S*_*m*_^3^) with the equality holding if and only if *G*_*H*_≅*S*_*m*_^3^.

**Proof**

Determine a vertex *v* of the maximum degree Δ as a root in *G*_*H*_, and let *k* ≥ 4 be an integer. Let *G*_*H*_*i*__ be the completely connected graph of 3-uniform linear hypertree *H*_*i*_ attached at *v*, and let *m*_*i*_ be the number of triangles of *G*_*H*_*i*__ for *i* = 1,2,…, Δ/2, respectively. We can repeatedly apply this transformation from Lemma 2 at some vertices whose degrees are not equal to two or 2*m*_*i*_ in *G*_*H*_*i*__ till *G*_*H*_*i*__ becomes a star. From Lemma 2, it satisfies that each application of this transformation strictly increases the number of closed walks and also increases Estrada index.

**Figure 4:**
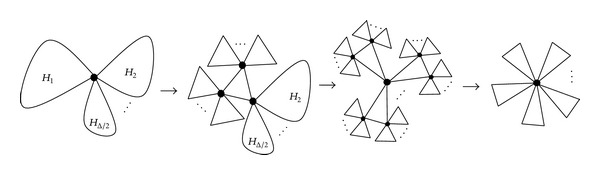
The procedure of transformation.

When all *G*_*H*_*i*__ turn into stars, we can again use Lemma 2 at the vertex *v* as long as there exists at least one vertex whose degree is not equal to two or 2∑*m*_*i*_, further increasing the number of closed walks. In the end of this procedure, we get the star *S*_*m*_^3^. The whole procedure of transformation is shown in Figure 4.

**Lemma 4 (see [20]).**

Let *v* be a vertex of a graph *G*, *G* − {*v*} = *G* − *v* for *v* ∈ *V*(*G*), and *C*(*v*) the set of cycles containing *v*. Consider

(9) P(G,λ)=λ·P(G−v,λ)−∑vw∈E(G)P(G−v−w,λ) −2∑Z∈C(v)P(G−V(Z),λ),

where *P*(*G* − *v* − *w*, *λ*) = 1 if *G* is a single edge and *P*(*G* − *V*(*Z*), *λ*) = 1 if *G* is a cycle.

Now, we calculate *EE*(*S*_*m*_^3^). Applying Lemma 4, we have

(10) $$\begin{array}{c} P\left(S_{m}^{3},\lambda \right)={\left(\lambda +1\right)}^{\left(n-1\right)/2}{\left(\lambda -1\right)}^{(n-3)/2}\left(\lambda^{2}-\lambda -n+1\right). \end{array}$$

By some simple calculating, we achieve the following eigenvalues:

(11) λ1=λ2=⋯=λ(n−1)/2=−1,λ(n+1)/2=λ(n+3)/2=⋯=λn−2=1,λn−1=1−4n−32,  λn=1+4n−32.

Then, we obtain

(12) EE(Sm3)=(n−1)2e+(n−3)2e +e(1+4n−3)/2+e(1−4n−3)/2.

Theorem 3 shows that the star *S*_*m*_^3^ has the maximum Estrada index in set Γ(*n*, *m*). Thus, according to previous definition, it is easy to show that the 3-uniform star *S*_*m*_^3^ has the maximum Estrada index among the set of 3-uniform linear hypertrees; that is,

(13) $$\begin{array}{c} EE\left(H\right)\leq EE\left(S_{m}^{3}\right), \end{array}$$

where

(14) EE(Sm3)=(n−1)2e+(n−3)2e +e(1+4n−3)/2+e(1−4n−3)/2.
